# ^177^Lu-trastuzumab radionuclide therapy: a promising strategy for trastuzumab-resistant brain metastases in HER2+ breast cancer

**DOI:** 10.1186/s43556-026-00573-7

**Published:** 2026-09-14

**Authors:** Liliana Santos, Ivanna Hrynchak, José Sereno, Hugo R.S. Ferreira, Magda Silva, Alexandra Fonseca, Rui Almeida, Paulo Teixeira, Antero J. Abrunhosa, Célia M. F. Gomes

**Affiliations:** 1 Faculty of Medicine, Institute of Pharmacology and Experimental Therapeutics, Coimbra Institute for Clinical and Biomedical Research (iCBR), University of Coimbra, Coimbra, Portugal; 2 Center for Innovative Biomedicine and Biotechnology (CIBB), University of Coimbra, Coimbra, Portugal; 3 Coimbra Institute for Biomedical Imaging and Translational Research, Institute for Nuclear Sciences Applied to Health (CIBIT/ICNAS), University of Coimbra, Coimbra, Portugal; 4 Clinical Academic Center of Coimbra (CACC), Coimbra, Portugal; 5 Pathology Department, Centro Hospitalar e Universitário de Coimbra, Coimbra, Portugal

**Keywords:** HER2 + breast cancer, Brain metastasis, Trastuzumab resistance, Targeted radionuclide therapy, Lutetium-177

## Abstract

Brain metastases (BrM) are a frequent and devastating complication of HER2-positive (HER2+) breast cancer (BC), affecting up to 30% of patients with metastatic disease. Although trastuzumab has transformed HER2+ BC treatment, its efficacy against BrM remains limited by brain-specific resistance mechanisms and heterogeneous blood–brain barrier (BBB) permeability. Here, we investigated whether HER2-targeted radionuclide therapy (TRT) using the β-emitting radioimmunoconjugate [^177^Lu]Lu-DOTA-trastuzumab could overcome trastuzumab resistance in HER2+ brain metastatic lesions. Brain-tropic cells retained HER2 expression but showed reduced sensitivity to trastuzumab, indicating the emergence of resistance mechanisms independent of target loss. By contrast, treatment with [^177^Lu]Lu-DOTA-trastuzumab produced robust DNA double-strand break-mediated cytotoxicity irrespective of trastuzumab sensitivity, demonstrating that resistance to antibody-mediated signaling inhibition does not confer cross-resistance to radiation-induced cell killing. In vivo, a single dose of [^177^Lu]Lu-DOTA-trastuzumab markedly reduced tumor progression and led to complete remission of established BrM in 40% of treated animals, whereas unconjugated trastuzumab showed minimal therapeutic benefit. Importantly, treatment was not associated with detectable neurotoxicity. While dynamic contrast-enhanced MRI demonstrated widespread BBB disruption across metastatic lesions, [^89^Zr]Zr-DFO-trastuzumab PET revealed marked heterogeneity in intracranial antibody delivery, with tracer uptake restricted to a subset of lesions. Collectively, these findings demonstrate that HER2-TRT can overcome trastuzumab resistance and eradicate brain metastatic lesions. Moreover, immuno-PET provides a non-invasive biomarker of intracranial antibody accessibility, supporting a theranostic strategy that combines patient stratification with TRT for HER2+ BrM.

**Supplementary Information:**

The online version contains supplementary material available at https://doi.org/10.1186/s43556-026-00573-7.

## Introduction

Breast cancer (BC) is the most commonly diagnosed cancer in women worldwide. Human epidermal growth factor receptor 2 (HER2) amplification occurs in approximately 20%–25% of metastatic BC cases and defines a clinically aggressive subtype associated with unfavorable outcomes [1]. The introduction of the anti-HER2 monoclonal antibody (mAb) trastuzumab has substantially improved the treatment of metastatic HER2-positive (HER2+) BC and contributed to prolonged patient survival [2]. However, improvements in systemic disease control have not eliminated the substantial risk of central nervous system (CNS) metastasis in patients with HER2+ disease [3].

Brain metastases (BrM) develop in up to 30% of patients with HER2+ metastatic BC and are associated with substantial morbidity and reduced survival [4]. Their management remains particularly challenging, as HER2-targeted therapies often exhibit limited efficacy within the CNS. This therapeutic failure has traditionally been attributed to restricted drug delivery across the blood–brain barrier (BBB), which limits the penetration of large molecules such as mAbs [5]. However, accumulating evidence indicates that BBB integrity is frequently compromised in metastatic lesions, allowing extravasation of macromolecules, including therapeutic antibodies [6, 7]. These observations suggest that impaired drug delivery alone cannot fully explain treatment failure in BrM. Indeed, increasing evidence points to the emergence of brain-specific resistance mechanisms that enable metastatic cells to survive despite adequate therapeutic exposure [8]. Among these, aberrant activation of the PI3K/AKT signaling pathway has been identified as a major driver of resistance to HER2-targeted therapies in both intracranial and extracranial metastatic disease [9–11]. Together, these findings highlight the biological complexity of HER2+ BrM and underscore the need for therapeutic strategies that overcome resistance mechanisms independent of downstream signaling blockade.

Targeted radionuclide therapy (TRT) represents a promising alternative approach. By coupling tumor-targeting molecules to therapeutic radionuclides, TRT enables the selective delivery of cytotoxic radiation to cancer cells, resulting in irreparable DNA damage and cell death [12]. Importantly, because its antitumor activity relies on radiation-induced cytotoxicity rather than inhibition of oncogenic signaling pathways, TRT may circumvent mechanisms that confer resistance to conventional targeted therapies [13]. The therapeutic potential of HER2-TRT has been supported by both preclinical and clinical studies in metastatic BC. Among the agents investigated, β-emitting radiopharmaceuticals such as [^177^Lu]Lu-DOTA-trastuzumab [14] and [^177^Lu]Lu-DOTA-ABY-027, a second-generation HER2-targeting affibody molecule [15], have shown encouraging antitumor activity. Lutetium-177 is especially attractive for this purpose due to its favorable physical characteristics, including a half-life of 6.65 days and a tissue penetration range of approximately 1–2 mm, enabling effective tumor irradiation while minimizing damage to surrounding healthy tissues [16].

Complementary molecular imaging studies using trastuzumab labeled with zirconium-89 (^89^Zr), indium-111 (^111^In), or copper-64 (^64^Cu) have demonstrated delivery to and accumulation within HER2+ BrM, indicating that at least a subset of brain metastatic lesions remains accessible to trastuzumab-based approaches [17–21]. Nevertheless, whether trastuzumab-based TRT can effectively eradicate HER2+ BrM and overcome resistance to conventional trastuzumab therapy remains poorly characterized.

Here, we show that [^177^Lu]Lu-DOTA-trastuzumab effectively eliminates trastuzumab-resistant HER2+ brain metastatic cells both in vitro and in vivo. We further demonstrate that lesion-specific therapeutic response is largely determined by heterogeneous vascular permeability and antibody accessibility, which can be non-invasively assessed using [^89^Zr]Zr-DFO-trastuzumab PET. Collectively, our findings establish HER2-TRT as a promising therapeutic option for trastuzumab-refractory BrM and support the development of a precision theranostic approach integrating immuno-PET-guided patient stratification with TRT.

## Results

### Brain-tropic BC cells acquire trastuzumab resistance while maintaining HER2 expression

Limited responsiveness to trastuzumab remains a persistent therapeutic challenge in metastatic HER2+ BC, particularly among patients with BrM. To determine whether adaptation to the brain microenvironment is associated with trastuzumab resistance, we compared parental HER2+ BC cell lines with their brain-tropic variants generated by serial in vivo selection.

**Fig. 1:**
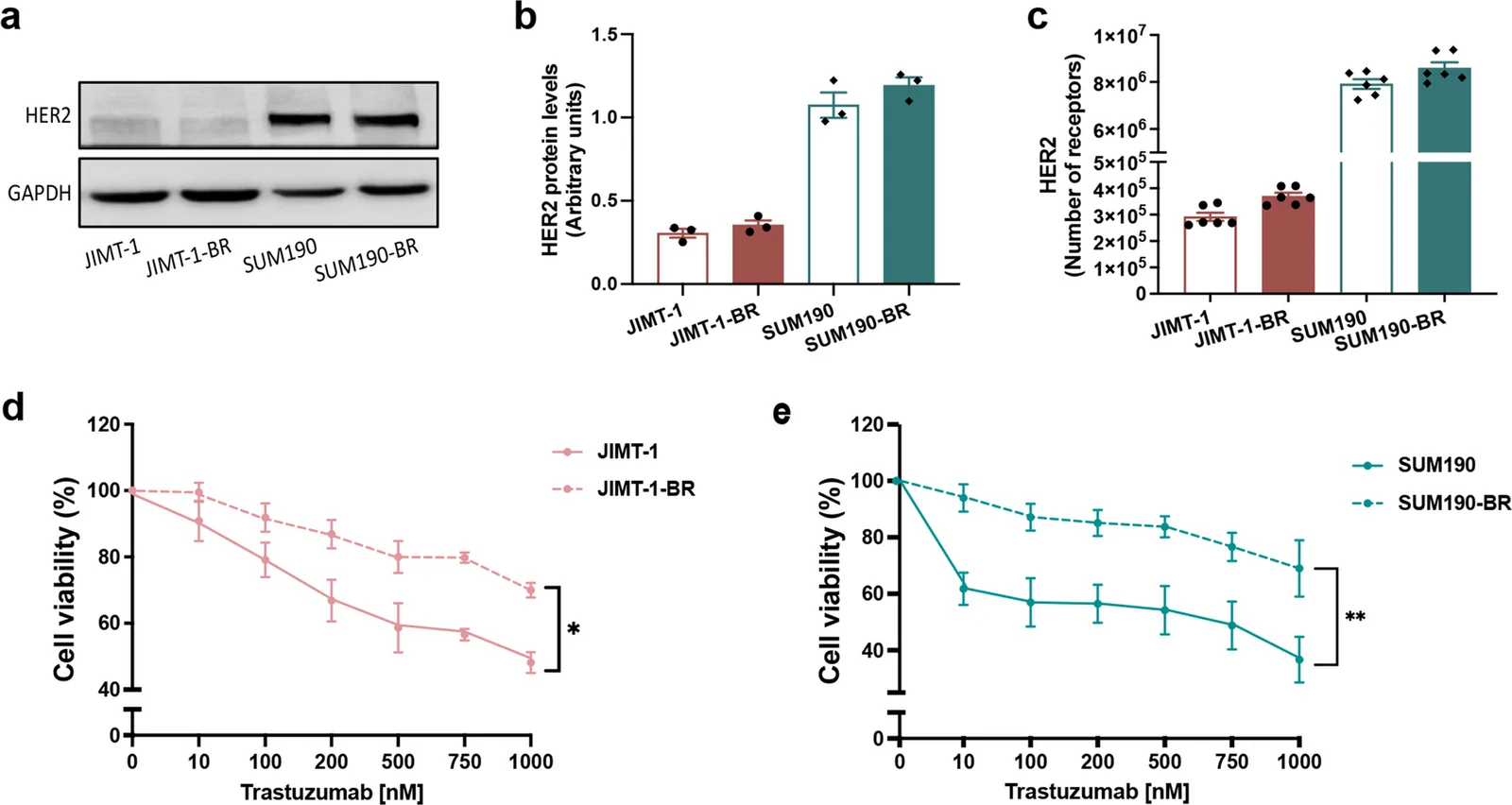
Brain metastatic HER2+ BC cells exhibit reduced sensitivity to trastuzumab while maintaining HER2 receptor density. **a** Representative western blot analysis of HER2 expression in parental (JIMT-1 and SUM190) and brain-tropic (JIMT-1-BR and SUM190-BR) BC cells. **b** Quantification of HER2 protein expression normalized to GAPDH (*n* = 3 independent experiments). **c** Quantification of cell-surface HER2 receptor density determined by flow cytometry (*n* = 6 independent experiments). Data are presented as individual values with mean ± SEM. **d-e** Concentration–response curves showing cell viability following treatment with increasing concentrations of trastuzumab in parental and brain metastatic cells (*n* = 6 independent experiments). Data are presented as mean ± SEM. *p < 0.05, **p < 0.01 compared with the respective parental cell line. Statistical significance was assessed using two-way ANOVA followed by Tukey’s multiple comparison test

We evaluated HER2 expression by Western blot analysis and flow cytometry (Fig. 1a-c). As expected, HER2 expression was more pronounced in SUM190 cells than in JIMT-1 cells, with mean surface receptor densities of 7.92 × 10⁶ and 2.92 × 10^5^ receptors per cell, respectively. However, total HER2 expression and cell-surface receptor density did not differ between parental and brain-tropic derivatives, indicating that HER2 expression is maintained during adaptation to the CNS.

Although HER2 expression remained high in the brain-tropic derivatives, these cells exhibited a markedly reduced response to trastuzumab compared with the corresponding parental lines (Fig. 1d-e). Across all tested concentrations, JIMT-1-BR and SUM190-BR cells showed higher viability, indicating that reduced trastuzumab sensitivity emerges during adaptation to the brain microenvironment despite retained HER2 expression. These findings suggest that HER2 remains a viable therapeutic target despite acquired resistance, supporting evaluation of alternative HER2-targeted strategies.

### Radiolabeled trastuzumab conjugates exhibit high radiochemical purity and stability

To investigate the potential of HER2-TRT in overcoming trastuzumab resistance, trastuzumab was functionalized with p-SCN-Bn-deferoxamine (DFO) to enable subsequent labeling with ^89^Zr, while p-SCN-Bn-1,4,7,10-tetraazacyclododecane-1,4,7,10-tetraacetic acid (DOTA) was used as the chelator for ^177^Lu labeling.

Successful chelator conjugation was verified by MALDI-TOF mass spectrometry, yielding mean chelator-to-antibody ratios of 7.5 for DFO and 6.3 for DOTA. Subsequent radiolabeling with [^89^Zr]Zr-oxalate or [^177^Lu]LuCl₃ produced [^89^Zr]Zr-DFO-trastuzumab and [^177^Lu]Lu-DOTA-trastuzumab, respectively, both achieving 100% radiochemical purity as determined by radio-TLC and SEC-HPLC (Fig. S1a–b). Importantly, both radioimmunoconjugates remained highly stable in PBS and mouse serum at 37 °C throughout the study (Fig. S1c).

### [^177^Lu]Lu-DOTA-trastuzumab induces DNA damage and cytotoxic effects irrespective of trastuzumab sensitivity status

**Fig. 2:**
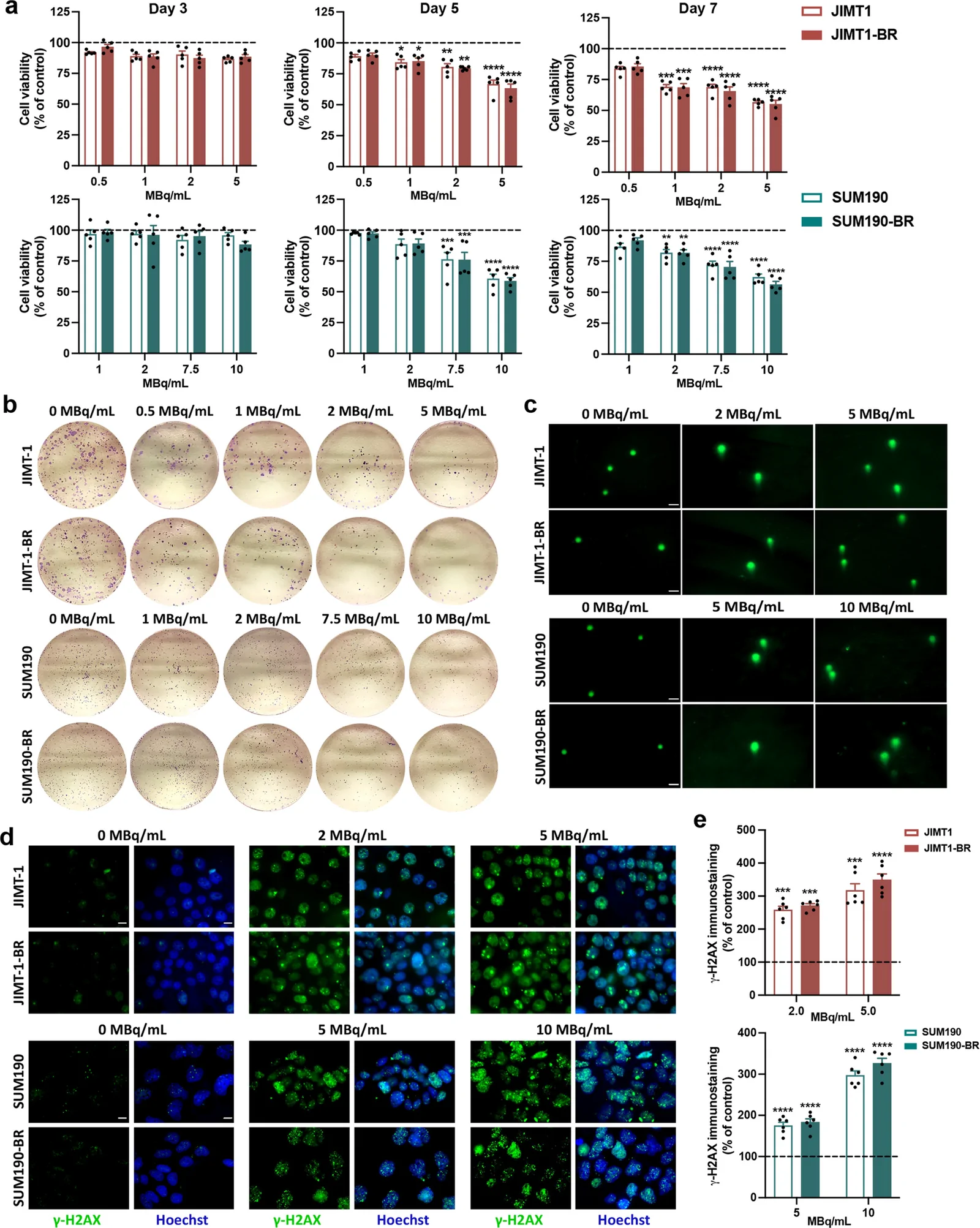
Parental and brain metastatic HER2+ BC cells exhibit comparable sensitivity to [^177^Lu]Lu-DOTA-trastuzumab-induced cytotoxicity and DNA damage. **a** Cell viability of parental and brain metastatic BC cells following incubation with increasing activities of [^177^Lu]Lu-DOTA-trastuzumab for 3, 5, and 7 days (*n* = 5 independent experiments). **b** Representative clonogenic assays showing colony formation 10–14 days following exposure to increasing activities of [^177^Lu]Lu-DOTA-trastuzumab (*n* = 3 independent experiments). **c** Representative comet assay images illustrating DNA damage in parental and brain metastatic cells 5 days after treatment with different activities of [^177^Lu]Lu-DOTA-trastuzumab (*n* = 3 independent experiments). Scale bar: 50 µm. **d** Representative immunofluorescence images of γ-H2AX foci staining (green) and nuclei counterstained with Hoechst 33342 (blue) in parental and brain metastatic cells 5 days after treatment with different activities of [^177^Lu]Lu-DOTA-trastuzumab. **e** Quantification of γ-H2AX immunostaining (*n* = 6 independent experiments). Statistical significance was determined using two-way ANOVA followed by Tukey’s multiple comparison test. Data are presented as individual values with mean ± SEM. *p < 0.05, **p < 0.01, ***p < 0.001, ****p < 0.0001 versus untreated controls (dashed line). Nuclei were counterstained with Hoechst 33342 (blue). Scale bar: 20 µm

We next investigated whether HER2-TRT could overcome the resistance observed in brain-tropic cells. Binding studies confirmed that radiolabeling preserved HER2 binding specificity, with [^89^Zr]Zr-DFO-trastuzumab showing selective uptake in HER2+ brain-tropic cells and negligible binding to HER2-negative MDA-MB-231 cells (Fig. S2). Consistent with HER2 surface receptor density quantified by flow cytometry, SUM190-BR cells exhibited approximately two-fold greater radioconjugate uptake than JIMT-1-BR cells. The antitumor effects of [^177^Lu]Lu-DOTA-trastuzumab were evaluated in parental and brain-tropic derivatives of both cell lines. Exposure to the radioimmunoconjugate progressively decreased cell viability in a concentration- and time-dependent manner, with the strongest cytotoxic response observed on day 7 post-treatment (Fig. 2a). Both variants displayed comparable sensitivity, indicating that acquired trastuzumab resistance does not impair responsiveness to HER2-TRT. Notably, JIMT-1 and JIMT-1-BR cells remained responsive despite their lower HER2 expression than SUM190-derived cells, demonstrating efficacy across a broad range of HER2 expression levels.

Consistent with these findings, clonogenic assays demonstrated a marked dose-dependent reduction in colony-forming capacity in all cell lines (Fig. 2b), with colony numbers progressively decreasing as [^177^Lu]Lu-DOTA-trastuzumab activity increased (Fig. S3a). To elucidate the mechanism responsible for the observed cytotoxicity, we quantified radiation-induced DNA double-strand breaks (DSBs) five days after treatment. Comet assays revealed significantly longer tail lengths in treated cells than in untreated controls, consistent with extensive radiation-induced DNA damage (Fig. 2c and Fig.S3b). Similarly, γ-H2AX analysis showed a pronounced increase in DSBs per nucleus following treatment (Fig. 2d). Parental and brain-tropic cells exhibited similar levels of DNA damage after treatment, suggesting that HER2-TRT maintains its ability to induce genotoxic stress despite the acquisition of reduced trastuzumab sensitivity.

Together, these findings establish [^177^Lu]Lu-DOTA-trastuzumab as an effective HER2-targeted therapy that induces DNA damage-mediated cytotoxicity irrespective of trastuzumab sensitivity, supporting its potential as a therapeutic approach for HER2+ BrM with limited sensitivity to conventional trastuzumab treatment.

### [^177^Lu]Lu-DOTA-trastuzumab suppresses the growth of trastuzumab-resistant HER2+ tumors

Following in vitro evaluation of therapeutic efficacy, we next investigated the antitumor activity of [^177^Lu]Lu-DOTA-trastuzumab in orthotopic HER2+ BC xenograft models derived from JIMT-1 and JIMT-1-BR cells, which showed comparable in vitro sensitivity to HER2-TRT. Although we initially selected JIMT-1-BR cells for their increased brain metastatic tropism, we implanted them into the mammary fat pad to establish primary tumors. Accordingly, all analyses in this section refer to the orthotopic primary tumor models.

**Fig. 3:**
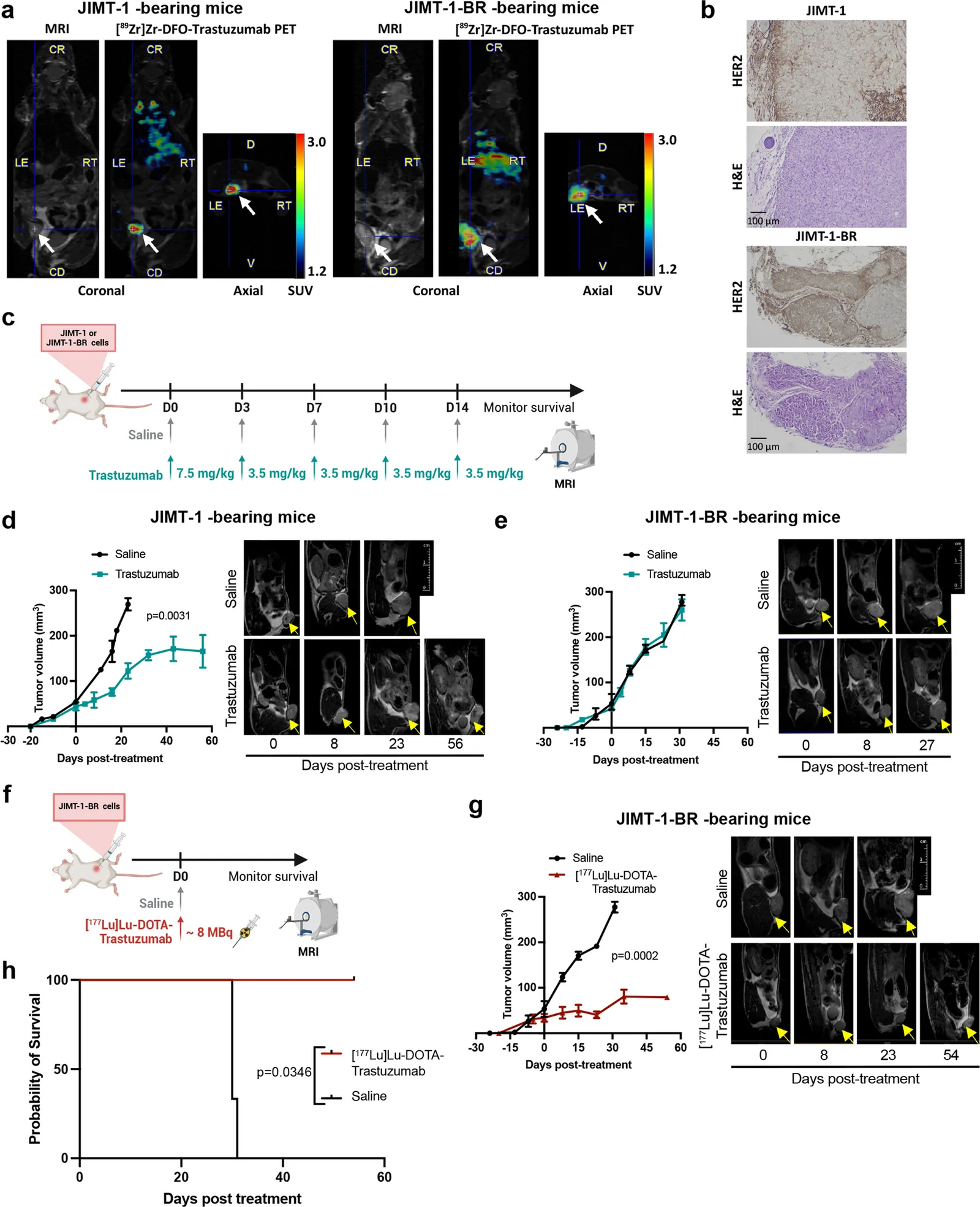
HER2-targeted radionuclide therapy overcomes trastuzumab resistance in primary HER2+ breast tumor models **a** Representative coronal and axial PET/MRI images acquired 5 days after intravenous administration of [^89^Zr]Zr-DFO-trastuzumab in mice bearing JIMT-1 (left) or JIMT-1-BR (right) mammary tumors. The color scale represents standardized uptake values (SUV) ranging from low (black) to high (red). Arrows indicate tumor locations. **b** Representative immunohistochemical staining for HER2 (top) and H&E staining (bottom) in JIMT-1 and JIMT-1-BR tumors. **c** Experimental design for trastuzumab treatment of JIMT-1 and JIMT-1-BR tumor-bearing mice. Mice received saline or trastuzumab (loading dose: 7.5 mg/kg on day 0; maintenance dose: 3.5 mg/kg on days 3, 7, 10, and 14), and MRI longitudinally monitored tumor progression. Tumor growth curves (left) and representative MRI images (right) of mice bearing **d** JIMT-1 or **e** JIMT-1-BR tumors following trastuzumab treatment (*n* = 3 independent animals per group). **f** Schematic representation of the treatment protocol for JIMT-1-BR tumor-bearing mice treated with a single intravenous dose of [^177^Lu]Lu-DOTA-trastuzumab (~8 MBq) or saline control. **g** Tumor growth curves (left) and representative MRI images (right) of mice bearing JIMT-1-BR tumors following [^177^Lu]Lu-DOTA-trastuzumab treatment. Arrows indicate tumor locations. **h** Kaplan–Meier survival analysis in mice treated with [^177^Lu]Lu-DOTA-trastuzumab (*n* = 3 independent animals per group). Data are presented as mean ± SEM. Statistical significance was assessed using two-way ANOVA followed by Bonferroni’s multiple comparison test for tumor growth analyses and the log-rank (Mantel–Cox) test for survival analysis

To confirm HER2 expression in vivo, mice underwent PET imaging following administration of [^89^Zr]Zr-DFO-trastuzumab. Prominent tracer accumulation was observed in both JIMT-1 and JIMT-1-BR tumors 5 days after injection (Fig. 3a), demonstrating selective targeting of HER2+ tumors. Quantitative PET analysis showed comparable tracer uptake in JIMT-1 and JIMT-1-BR tumors (SUV_mean_ 2.70 and 2.75, respectively, Fig. S4a). These findings were corroborated by immunohistochemistry, which showed intense, complete membrane staining in ≤ 10% of tumor cells, corresponding to an equivocal HER2 immunohistochemistry score (2+) in both tumor types (Fig. 3b). Biodistribution studies demonstrated preferential tumor uptake, with additional activity in the liver and spleen, consistent with hepatobiliary clearance of intact IgG antibodies (Fig. S4b). Bone uptake was also observed, likely reflecting the accumulation of free ^89^Zr following radiolysis or metabolic degradation of the radioconjugate, as previously reported [22]. To determine whether brain tropism alters responsiveness to trastuzumab in vivo, mice bearing either JIMT-1 or JIMT-1-BR tumors were treated with trastuzumab or saline once tumors reached approximately 45 mm^3^ (*n* = 3 per group; Fig. 3c), following a previously established treatment regimen [23]. In mice bearing parental JIMT-1 tumors, trastuzumab treatment markedly restrained tumor progression, extending the time to reach the predefined endpoint by 33 days compared with saline controls (*p* = 0.0031, ηp^2^ = 0.953; Fig. 3d). By day 23, tumors in control mice had exceeded 250 mm^3^, whereas trastuzumab-treated tumors remained below 170 mm^3^ throughout the 56-day monitoring period, as assessed by MRI. In contrast, JIMT-1-BR tumors showed no appreciable response to trastuzumab, with growth trajectories comparable to those of saline-treated mice and tumor volumes exceeding 250 mm^3^ between days 23 and 31 (Fig. 3e). These findings demonstrate that the trastuzumab-resistant phenotype of JIMT-1-BR cells observed in vitro is maintained in vivo.

We next investigated whether HER2-TRT could overcome the trastuzumab-resistant phenotype (Fig. 3f). In mice bearing JIMT-1-BR tumors, a single dose of [^177^Lu]Lu-DOTA-trastuzumab (~8 MBq) significantly delayed tumor progression by 23 days compared with saline-treated controls (*p* = 0.0002, ηp^2^ = 0.96; Fig. 3g). In a subset of treated animals, tumor growth resumed after approximately 35 days; however, tumor volumes subsequently stabilized at 65–95 mm^3^, indicating sustained tumor control rather than complete regression. Consistent with these findings, HER2-TRT significantly improved survival, with all treated mice surviving to the end of the 56-day observation period, whereas all control animals reached the study endpoint approximately 32 days after treatment (*p* = 0.0346; Fig. 3h). Importantly, treatment was not associated with significant body weight loss, supporting its overall tolerability.

Together, these findings demonstrate that [^177^Lu]Lu-DOTA-trastuzumab provides durable tumor control in HER2+ tumors refractory to conventional HER2-targeted therapy, supporting its further evaluation as a therapeutic strategy for trastuzumab-resistant HER2+ disease.

### BrM display heterogeneous BBB permeability and variable accessibility to trastuzumab

Brain metastatic lesions undergo extensive but structurally aberrant vascular remodeling, characterized by disrupted tight junctions, altered pericyte coverage, and increased endothelial fenestration [7]. These alterations are spatially heterogeneous, resulting in variable BBB permeability that may differentially influence drug delivery across metastatic lesions [24].

**Fig. 4:**
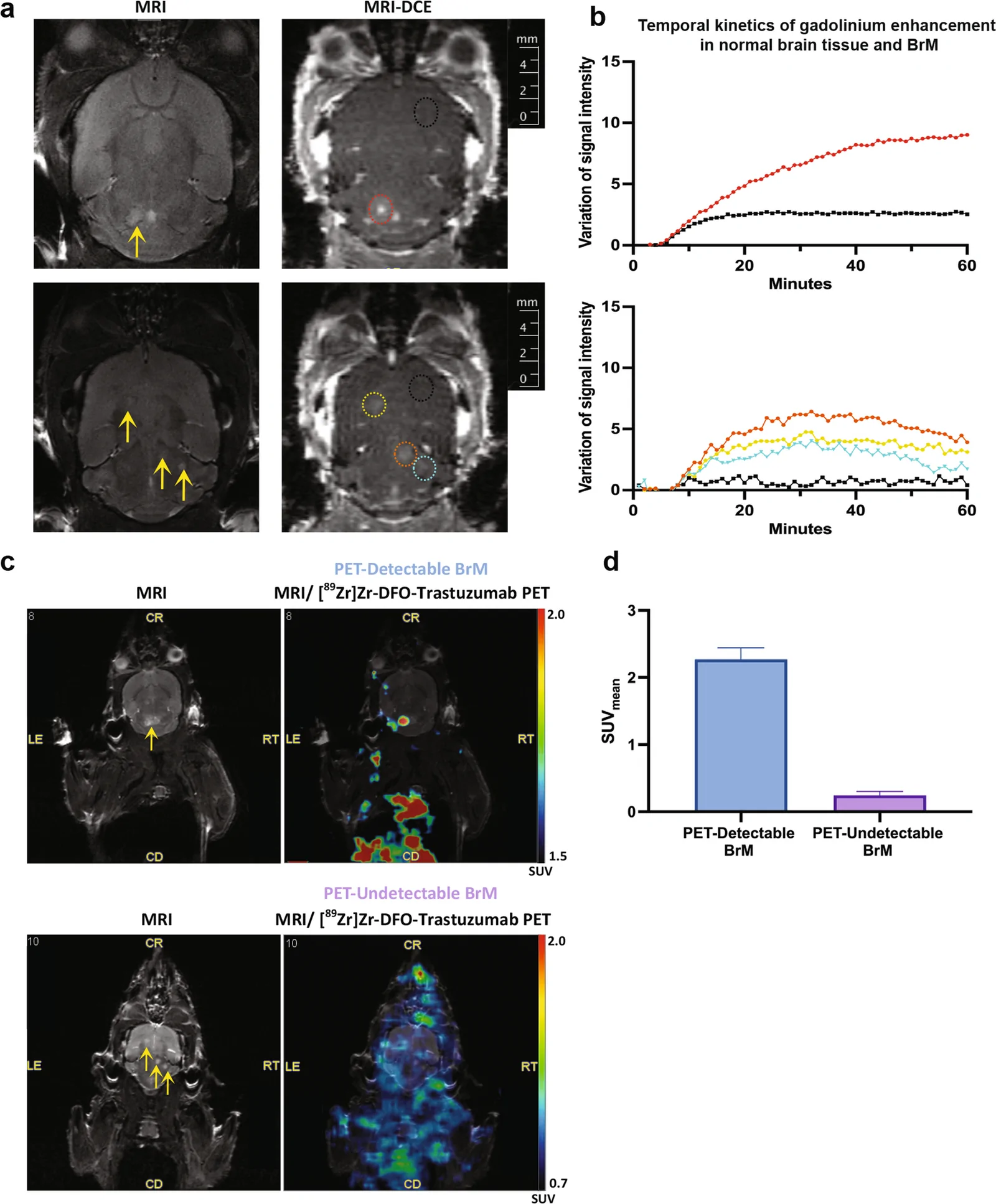
Brain metastatic lesions exhibit heterogeneous BBB permeability and variable trastuzumab accessibility. **a** Representative anatomical MRI images (left) and corresponding dynamic contrast-enhanced MRI (DCE-MRI) images (right) of mice bearing HER2+ BrM. Yellow arrows indicate metastatic lesions. Colored circles denote the regions of interest (ROIs) corresponding to individual metastatic lesions, while black circles indicate corresponding contralateral non-tumor brain regions used as controls. **b** Quantification of Gadolinium extravasation over time for the individual metastatic lesions (colored circles) and non-tumor brain regions (black circles) indicated in **a**. Each curve corresponds to the ROI marked by the circle of the same color in **a**. **c** Representative PET/MRI images acquired 5 days after intravenous administration of [^89^Zr]Zr-DFO-trastuzumab in mice bearing BrM (*n* = 5 independent animals). The color scale represents standardized uptake values (SUV) ranging from low (black) to high (red). Yellow arrows indicate brain metastatic lesions. **d** SUV_mean_ of [^89^Zr]Zr-DFO-trastuzumab measured in MRI-detectable brain metastatic lesions with detectable radiotracer uptake and in those without detectable radiotracer uptake

To characterize BBB permeability, DCE-MRI with gadolinium was performed after intracranial lesions became detectable by MRI (20–30 days after tumor cell injection; mean volume of 0.33 mm^3^). Gadolinium extravasation was observed in all animals (*n* = 5), indicating that BBB disruption is a consistent feature of metastatic lesions. However, contrast enhancement varied substantially among lesions, ranging from pronounced and sustained accumulation to only modest enhancement (Fig. 4a-b). Gadolinium accumulation was not observed in non-tumor brain regions, supporting the preservation of BBB integrity in healthy tissue.

To evaluate whether BBB disruption was sufficient to allow intracranial delivery of a full-length therapeutic antibody, the same animals subsequently underwent immuno-PET imaging with [^89^Zr]Zr-DFO-Trastuzumab. PET scans were obtained 5 days after tracer administration, which corresponds to the optimal imaging window for antibody biodistribution and tumor uptake.

Despite the widespread vascular permeability detected by DCE-MRI, intracranial accumulation of [^89^Zr]Zr-DFO-Trastuzumab was observed in only two of five animals and was restricted to a subset of metastatic lesions (Fig. 4c). This revealed a striking dissociation between the extravasation of a low-molecular-weight contrast agent and the delivery of a full-length therapeutic antibody. Quantitative PET analysis further demonstrated substantial inter-lesional heterogeneity, with SUV_mean_ values reaching 2.3 in tracer-positive lesions but remaining as low as 0.25 in others (Fig. 4d).

These findings indicate that BBB disruption detected by DCE-MRI does not necessarily translate into therapeutic antibody penetration, establishing vascular permeability as an unreliable surrogate for intracranial antibody accessibility.

Collectively, these results demonstrate that BrM exhibit marked heterogeneity in therapeutic accessibility. Although small-molecule contrast agents readily extravasate within metastatic lesions, the delivery of full-length antibodies remains restricted to a subset of tumors, even in the context of widespread BBB disruption. Consequently, [^89^Zr]Zr-DFO-trastuzumab immuno-PET offers a more functionally relevant assessment of intracranial antibody accessibility than DCE-MRI and may represent a clinically actionable imaging biomarker to predict the delivery of HER2-targeted therapies to BrM. These results findings support the integration of immuno-PET into a theranostic framework to guide patient stratification and optimize treatment selection.

### Therapeutic efficacy of [^177^Lu]Lu-DOTA-trastuzumab in HER2+ BrM

**Fig. 5:**
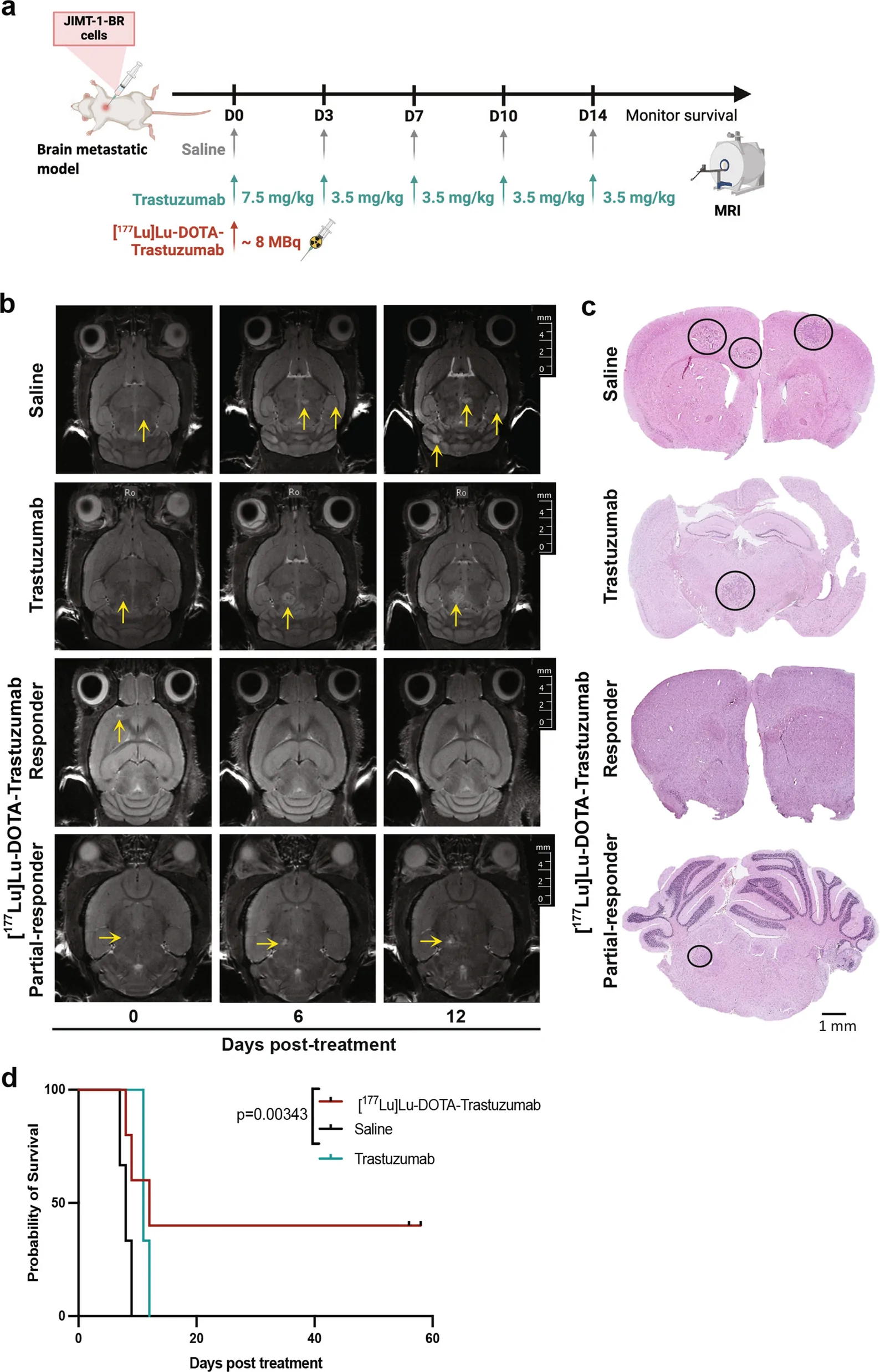
[^177^Lu]Lu-DOTA-trastuzumab induces regression of established HER2+ BrM and prolongs survival. **a** Experimental design of the treatment protocol for mice bearing JIMT-1-BR BrM. Animals received saline (*n* = 3 independent animals), trastuzumab (loading dose: 7.5 mg/kg on day 0; maintenance dose: 3.5 mg/kg on days 3, 7, 10, and 14; *n* = 3 independent animals), or a single intravenous administration of [^177^Lu]Lu-DOTA-trastuzumab (~8 MBq; *n* = 5 independent animals). MRI longitudinally monitored brain metastatic progression. **b** Representative MRI images illustrating the progression or regression of brain metastatic lesions in the different treatment groups over time. Yellow arrows indicate brain metastatic lesions. **c** Representative H&E-stained whole-brain sections confirming the presence or absence of metastatic lesions following treatment. Scale bars: 1 mm. **d** Kaplan–Meier survival analysis in mice treated with [^177^Lu]Lu-DOTA-trastuzumab compared with saline-treated controls. Statistical significance was assessed using the log-rank (Mantel–Cox) test

Next, we evaluated the therapeutic efficacy of trastuzumab-based TRT in an independent cohort of mice bearing established BrM. Following MRI confirmation of intracranial lesions, animals received a single intravenous administration of [^177^Lu]Lu-DOTA-trastuzumab (*n* = 5, ~ 8 MBq), unconjugated trastuzumab (*n* = 3), or saline (*n* = 3). Tumor burden was monitored longitudinally by MRI at 4-day intervals, enabling quantitative evaluation of metastatic progression over time (Fig. 5a).

Consistent with the findings in primary tumors, unconjugated trastuzumab failed to induce a measurable therapeutic response in brain metastatic lesions. Treated animals exhibited progressive intracranial disease comparable to that of saline-treated controls, confirming the limited efficacy of conventional HER2 signaling blockade in this setting. By comparison, a single dose of [^177^Lu]Lu-DOTA-trastuzumab produced a pronounced therapeutic response. Complete remission was achieved in two of five treated animals (40%), as demonstrated by the sustained absence of detectable lesions on serial MRI and confirmed by histopathological analysis (Fig. 5b-c). Among the remaining animals, two exhibited partial responses characterized by delayed metastatic progression relative to trastuzumab- and saline-treated groups, whereas only one animal showed progressive disease. These results demonstrate that HER2-TRT can induce durable tumor control in established BrM. Importantly, treatment was not associated with body weight loss or detectable neurotoxicity. γH2AX immunostaining demonstrated DNA damage selectively within metastatic lesions, whereas no evident γH2AX immunoreactivity was observed in the surrounding brain parenchyma (Fig. S5a). Consistent with these findings, Cresyl Violet staining showed preserved tissue architecture and neuronal morphology in adjacent brain tissue, with no evidence of neuronal loss, necrosis, or other histopathological abnormalities following [^177^Lu]Lu-DOTA-trastuzumab administration (Fig. S5b). Collectively, these findings support a favorable safety profile for HER2-TRT in this preclinical model. Although therapeutic responses varied between animals, treatment with [^177^Lu]Lu-DOTA-trastuzumab significantly extended overall survival relative to saline-treated controls (*p* = 0.00343; Fig. 5d).

The heterogeneous response pattern is consistent with the marked inter- and intra-lesional variability in BBB permeability and HER2-targeted antibody uptake identified by DCE-MRI and immuno-PET, indicating that lesion-specific radiopharmaceutical delivery is a major determinant of therapeutic efficacy.

Together, these findings demonstrate that HER2-TRT is an effective therapeutic strategy for trastuzumab-resistant BrM and establish immuno-PET as a non-invasive imaging approach to assess lesion-specific radiopharmaceutical delivery.

The comparative therapeutic outcomes across all experimental models are summarized in Table 1.

**Table 1:** Therapeutic outcomes of trastuzumab and [^1^⁷⁷Lu]Lu-DOTA-trastuzumab in HER2+ primary tumors and BrM

Experimental Model	Treatment	Response rate	Tumor Progression	Time to Endpoint (days)
Primary tumor JIMT-1	Saline	0/3	Progressive disease	23
	Trastuzumab	3/3	Tumor growth delay (+33 days)	> 55
Primary tumor JIMT-1-BR	Saline	0/3	Progressive disease	27
	Trastuzumab	0/3	Progressive disease	27
	[^177^Lu]Lu-DOTA-Trastuzumab	3/3	Tumor growth delay (+23 days)	> 55
Brain metastases JIMT-1-BR	Saline	0/3	Progressive disease	8
	Trastuzumab	0/3	Progressive disease	11
	[^177^Lu]Lu-DOTA-Trastuzumab	4/5	Complete remission (2/5) and delayed progression (2/5)	> 55 (2/5)

## Discussion

Despite major advances in HER2-targeted therapies, BrM remains one of the most challenging complications of HER2+ BC. Although improved systemic disease control has significantly extended patient survival, the incidence of CNS metastases remains disproportionately high in this molecular subtype. Historically, the limited efficacy of HER2-directed therapies in the brain has been attributed to restricted drug delivery across BBB. However, increasing evidence suggests that impaired BBB penetration does not fully explain therapeutic failure [9].

Here, we show that tumor cell adaptation to the brain microenvironment is associated with acquired trastuzumab resistance despite sustained HER2 expression. Both in vitro and in vivo, brain-tropic tumors remained refractory to trastuzumab while maintaining HER2 levels comparable to their parental counterparts, indicating that resistance results from adaptive molecular reprogramming rather than target loss.

These findings align with previous reports showing that exposure to the CNS microenvironment can trigger compensatory signaling mechanisms, such as HER2/HER3 heterodimer formation and subsequent activation of the PI3K/AKT and MAPK/ERK cascades, thereby reducing tumor cells' reliance on HER2-driven signaling [25–27]. Although not directly investigated in this study, our findings suggest that trastuzumab resistance arises from microenvironment-driven signaling rewiring rather than HER2 downregulation, thereby preserving HER2 as a viable target for alternative HER2-directed therapies, including antibody–drug conjugates and TRT.

Based on this rationale, we investigated whether HER2-TRT could overcome resistance to conventional HER2 blockade. Unlike trastuzumab, which inhibits receptor-mediated oncogenic signaling, [^177^Lu]Lu-DOTA-trastuzumab delivers β-emitting radiation directly to HER2-expressing cells, inducing irreparable DNA damage independently of upstream signaling pathways. Consistent with this mechanism, [^177^Lu]Lu-DOTA-trastuzumab induced marked DNA damage and cytotoxic effects in both parental and brain-tropic cells, retaining full efficacy in trastuzumab-resistant variants.

The therapeutic activity observed in vitro translated into robust antitumor effects in mice*.* A single administration of [^177^Lu]Lu-DOTA-trastuzumab significantly delayed intracranial tumor growth and prolonged survival, inducing complete remission of established BrM in 40% of treated animals, whereas unconjugated trastuzumab produced minimal therapeutic benefit. These findings underscore the fundamental mechanistic difference between receptor blockade and radiation-induced cytotoxicity. These results are consistent with previous preclinical studies reporting enhanced antitumor activity of [^177^Lu]Lu-DOTA-trastuzumab over unconjugated trastuzumab [28, 29]. Furthermore, early clinical studies have demonstrated the safety and feasibility of this radiopharmaceutical in patients with HER2+ BC [14, 30], providing a strong translational rationale for its further evaluation in patients with CNS disease.

The β-particle cross-fire effect may further enhance therapeutic efficacy by irradiating adjacent tumor cells with limited radioconjugate uptake, thereby extending cytotoxic activity beyond cells directly targeted by the radioconjugate. This bystander effect is particularly advantageous in BrM, where heterogeneous BBB permeability and variable antibody delivery are likely to produce non-uniform intratumoral radioconjugate distribution.

Beyond demonstrating therapeutic efficacy, our study highlights intracranial antibody accessibility as an important determinant of treatment response. DCE-MRI demonstrated BBB disruption in all metastatic lesions, albeit to varying degrees, indicating that vascular permeability is a common feature of established BrM. However, [^89^Zr]Zr-DFO-trastuzumab immuno-PET revealed substantial heterogeneity in antibody delivery, with detectable tracer uptake restricted to a subset of lesions.

Similar discrepancies between MRI contrast enhancement and antibody uptake have been reported in both preclinical and clinical settings. Terrell-Hall et al*.* demonstrated that [^125^I]I-trastuzumab localizes to regions of BBB disruption within HER2+ brain lesions [24]. Likewise, Puttemans et al*.* reported heterogeneous uptake of [^111^In]In-trastuzumab across lesions with distinct vascular characteristics [23], while Kurihara et al*.* observed discordance between MRI-detectable lesions and [^64^Cu]Cu-trastuzumab PET/CT uptake in patients [18]. Collectively, these findings indicate that radiological evidence of BBB disruption does not necessarily predict antibody accessibility, supporting the concept that BBB permeability is dynamic and spatially heterogeneous rather than binary.

The heterogeneous immuno-PET signal likely reflects lesion-specific differences in vascular architecture and microenvironmental composition. Variations in endothelial integrity, pericyte coverage, extracellular matrix composition, and inflammatory signaling are likely contributors. For example, pericyte dysfunction disrupts PDGF-B/PDGFRβ signaling required for BBB maintenance, whereas local inflammatory cytokines promote endothelial fenestration and increased vascular permeability. Concurrent alterations in extracellular matrix composition further remodel the perivascular niche, collectively generating heterogeneous antibody accessibility across metastatic lesions [6, 7, 31]. This biological heterogeneity likely contributes to the variable therapeutic responses observed following HER2-TRT and may explain why complete responses were achieved only in a subset of animals. Collectively, these findings suggest that lesion-level accessibility may be as important as HER2 expression in determining therapeutic outcome, highlighting the need for quantitative imaging biomarkers to guide patient selection.

Although full-length trastuzumab provides a clinically validated theranostic platform, alternative HER2-targeting strategies are being explored to improve intracranial drug delivery. HER2-specific nanobodies have attracted considerable interest owing to their small size, favorable tissue penetration, and high target affinity [32]. Radiolabelled anti-HER2 nanobodies, including 2Rs15d, have demonstrated promising therapeutic activity in preclinical models of trastuzumab-resistant BrM [23]. Nevertheless, their rapid systemic clearance may require higher administered activities or repeated dosing, potentially limiting clinical translation.

Several limitations should be acknowledged. The in vivo studies were conducted in relatively small cohorts, limiting assessment of biological variability and inter-lesion response heterogeneity. Nevertheless, the consistency of the therapeutic effects across experimental models and the large treatment effect sizes recorded for the primary tumor outcomes support the robustness of our findings. Furthermore, only a single administration of [^177^Lu]Lu-DOTA-Trastuzumab was evaluated. Future studies should evaluate fractionated or repeated dosing regimens to determine whether therapeutic efficacy can be further enhanced.

In conclusion, our findings establish [^177^Lu]Lu-DOTA-trastuzumab as a promising therapeutic strategy to overcome trastuzumab resistance in HER2+ BC, with demonstrated activity against both primary tumors and BrM. Importantly, therapeutic efficacy within the CNS depends not only on HER2 expression but also on lesion-specific antibody accessibility. In this context, [^89^Zr]Zr-DFO-trastuzumab immuno-PET provides a non-invasive approach to assess intratumoral antibody delivery, assess target engagement, and support treatment stratification before therapy. By identifying lesions accessible to antibody-based therapeutics, immuno-PET could enable more precise patient selection, guide individualized treatment strategies, and ultimately improve the therapeutic index of HER2-TRT. Collectively, these findings support a clinically translatable theranostic framework integrating predictive molecular imaging with TRT for the management of HER2+ BrM.

## Materials and methods

### Preparation of [^89^Zr]Zr-DFO-trastuzumab

Trastuzumab (Herceptin; Evidentic GmbH) was conjugated to the bifunctional chelator p-SCN-Bn-DFO (Macrocyclics) as previously described [33]. Briefly, trastuzumab (2 mg/mL, 0.5 mL) was incubated with a 50-fold molar excess of DFO (25.95 mg/mL, 10 μL) for 1.5 h at room temperature at pH 9, followed by purification using PD-10 size-exclusion chromatography (GE Healthcare). The average number of DFO molecules per antibody was determined by MALDI–TOF mass spectrometry as previously described [34].

Zirconium-89 was produced via the ^89^Y(p,n)^89^Zr nuclear reaction using an IBA Cyclone 18/9 cyclotron equipped with an IBA Nirta liquid target. Irradiations were performed at 14 meV and 30–35 μA for 3–4 h. Following irradiation, ^89^Zr was purified using hydroxamate resin and eluted with oxalic acid.

Radiolabeling was performed by incubating neutralized [^89^Zr]Zr-oxalate (30–120 MBq) with DFO-trastuzumab (200–600 μg) in HEPES buffer (pH 6.5–7.5) for 60 min at 37 °C. The product was purified by PD-10 chromatography, and radiochemical purity (RCP) was assessed by radio-TLC and SEC-HPLC using a Superdex™ 200 10/300 GL column (GE Healthcare) on an Agilent™ 1260 Infinity system. Radio-TLC was quantified using a Raytest miniGita scanner and GinaStar software.

### Preparation of [^177^Lu]Lu-DOTA-trastuzumab

Trastuzumab (Herceptin; Evidentic GmbH) was conjugated to p-SCN-Bn-DOTA (Macrocyclics) as previously described [35]. Briefly, trastuzumab was incubated with a 20-fold molar excess of DOTA for 24 h at 37 °C and purified by PD-10 size-exclusion chromatography. Chelator-to-antibody ratios were determined by MALDI–TOF mass spectrometry [34]. For radiolabeling, [^177^Lu]LuCl₃ (ITM Medical Isotopes GmbH; 185–370 MBq) was incubated with DOTA-trastuzumab (200–600 μg) in ammonium acetate buffer for 30 min at 37 °C. The radioimmunoconjugate was purified by size-exclusion chromatography, and RCP was evaluated by radio-TLC and SEC-HPLC.

### Cell culture

Human HER2 + BC cell lines JIMT-1 and SUM190 and their corresponding brain-seeking derivatives, JIMT-1-BR and SUM190-BR, were kindly provided by Dr. Patricia Steeg (National Cancer Institute, Bethesda, MD, USA). Brain metastatic variants were established by serial intracardiac inoculation followed by isolation of metastatic cells from brain lesions, as previously reported for JIMT-1-BR [36] and SUM190-BR [7].

JIMT-1 and MDA-MB-231 cells were maintained in Dulbecco's Modified Eagle Medium (DMEM; Sigma-Aldrich) supplemented with 10% fetal bovine serum (FBS; Gibco) and 1% antibiotic–antimycotic solution (Gibco). SUM190 cells were cultured in Ham's F-12 medium (Sigma-Aldrich) supplemented with 1 μg/mL hydrocortisone (Sigma-Aldrich), 10 mM HEPES (Sigma-Aldrich), insulin-transferrin-selenium-ethanolamine supplement (Gibco), 10 nM triiodothyronine (Sigma-Aldrich), 1 g/L bovine serum albumin (BSA; Sigma-Aldrich), and 1% antibiotic–antimycotic (Gibco).

### Flow cytometry

JIMT-1 and SUM190 cells and their brain metastatic derivatives were collected as single-cell suspensions (2.5 × 10^5^ cells per sample), maintained on ice for 20–30 min, and centrifuged at 170 × g for 3 min. Cells were resuspended in 200 μL of ice-cold PBS containing 1% BSA, centrifuged again, and incubated with APC-conjugated anti-HER2 antibody (clone 24D2, BioLegend) in PBS-BSA for 45–60 min on ice. Cells were washed twice and analyzed using a BD FACSCalibur flow cytometer. Dead cells were excluded using 7-aminoactinomycin D (7-AAD; Sigma-Aldrich).

Cell-surface HER2 expression was quantified as Molecules of Equivalent Soluble Fluorochrome (MESF) using calibration curves generated with the Quantum™ MESF APC microsphere kit (Bangs Laboratories) and QuickCal v2.3 software. Antibody-binding capacity (ABC) was calculated by dividing MESF values by 1.33, corresponding to the fluorochrome-to-antibody labeling ratio. Assuming a 1:1 interaction between trastuzumab and HER2, ABC values were used to estimate cell-surface HER2 receptor density.

### Western blot

Whole-cell protein lysates were prepared using standard lysis buffer procedures, and equal amounts of protein were separated by SDS-PAGE and transferred to PVDF membranes (Bio-Rad). Membranes were blocked with 5% BSA in TBS-T and incubated overnight at 4 °C with primary antibodies against HER2 (1:1000; Cell Signaling Technology, Cat. No. 2165) and β-actin (1:1000; Cell Signaling Technology, Cat. No. 4967). After washing, membranes were incubated for 1 h at room temperature with the corresponding horseradish peroxidase-conjugated secondary antibody (1:10,000). Bands were detected using Clarity™ ECL Western Blotting Substrate (Bio-Rad), visualized with an ImageQuant LAS 500 chemiluminescence imaging system (GE Healthcare) and quantified using ImageJ software (National Institutes of Health).

### Cell viability assay

To assess trastuzumab sensitivity, JIMT-1 and SUM190 cells and their brain metastatic variants were seeded in 48-well plates at 1 × 10^3^ and 3.5 × 10^3^ cells/well, respectively, and allowed to attach overnight. Cells were treated with trastuzumab (10–1000 nM; Herceptin; Evidentic GmbH) for 5 days, washed twice with PBS, and maintained in drug-free culture medium for an additional 5 days. Cell viability was assessed using the resazurin reduction assay (Sigma-Aldrich), with fluorescence measured at 530/590 nm using a Synergy HT microplate reader (Biotek).

For [^177^Lu]Lu-DOTA-trastuzumab, JIMT-1 and SUM190 cells and their brain metastatic derivatives were seeded in 96-well plates at 4 × 10^3^ and 1 × 10^4^ cells/well, respectively. Following overnight attachment, JIMT-1 cells were incubated with 0.5, 1, 2, or 5 MBq/mL, whereas SUM190 cells received 1, 5, 7.5, or 10 MBq/mL of radioconjugate for 2 h at 37 °C. Untreated cells served as negative controls. After treatment, the medium was replaced and cells were incubated for 3, 5, or 7 days. Cell viability was quantified using the CellTiter-Glo® 3D Cell Viability Assay (Promega) according to the manufacturer's instructions, with luminescence recorded on a Synergy HT microplate reader (Biotek). Cell viability was expressed as a percentage relative to untreated controls. Experiments were independently repeated five times, with at least four technical replicates per condition.

### Colony formation assay

Cells were seeded in 12-well plates at 600 cells/well (JIMT-1) and 4 × 10^3^ cells/well (SUM190) and allowed to adhere overnight. JIMT-1 cells were treated with 0.5, 1, 2, or 5 MBq/mL and SUM190 cells with 1, 5, 7.5, or 10 MBq/mL [^177^Lu]Lu-DOTA-trastuzumab for 2 h at 37 °C. Untreated cells were included as controls. After medium replacement, cells were maintained under standard culture conditions for 10–14 days to allow colony formation. Colonies were washed with PBS, fixed in 1% formaldehyde for 15 min, stained with 0.1% crystal violet in ethanol for 15 min, and manually counted. Each condition was performed in duplicate and independently repeated three times.

### γ-H2AX assay

JIMT-1 and SUM190 cells and their brain metastatic derivatives were seeded onto glass coverslips in 24-well plates at 4.5 × 10^4^ and 1 × 10^5^ cells/well, respectively, and allowed to adhere overnight. Cells were treated with [^177^Lu]Lu-DOTA-trastuzumab for 2 h at 37 °C (JIMT-1: 2 or 5 MBq/mL; SUM190: 5 or 10 MBq/mL), followed by medium replacement and a further 5-day incubation. Untreated cells were included as controls. Cells were washed three times with PBS, fixed with 4% paraformaldehyde, permeabilized with 0.5% Triton X-100, and incubated overnight at 4 °C with anti-γ-H2AX (Ser139) antibody (1:250; Abcam, Cat. No. ab81299). Following incubation with FITC-conjugated anti-rabbit secondary antibody (1:200; Invitrogen), nuclei were counterstained with Hoechst 33342 (5 μg/mL) and mounted using VECTASHIELD mounting medium (Vector Laboratories). Images were acquired using a Carl Zeiss Axio Observer Z1 microscope with a 20 × objective. Six fields per coverslip were analyzed, with at least 20 nuclei quantified per condition using ImageJ software (National Institutes of Health). Experiments were performed in duplicate and independently repeated six times.

### Comet assay

JIMT-1 and SUM190 cells and their brain metastatic counterparts were seeded in 6-well plates at 1 × 10^5^ and 3 × 10^5^ cells/well, respectively, and allowed to attach overnight. Cells were treated with [^177^Lu]Lu-DOTA-trastuzumab for 2 h at 37 °C (JIMT-1: 2 or 5 MBq/mL; SUM190: 5 or 10 MBq/mL), followed by medium replacement and a further 5-day incubation. Untreated cells served as negative controls. Cells were collected and processed using the Comet Assay Kit (Enzo Life Sciences, Cat. No. ADI-900–166) according to the manufacturer's instructions. Images were acquired using a Carl Zeiss Axio Observer Z1 microscope, and DNA damage was quantified by measuring comet tail length with ImageJ software (National Institutes of Health). Eight fields per slide were analyzed. The assay was independently performed three times for each cell line.

### Animal studies

Female athymic Swiss FoxnI nu/nu mice (10–12 weeks old; 20–25 g) were obtained from ICNAS and maintained under specific pathogen-free conditions in individually ventilated cages. For the orthotopic BC model, mice received 2 × 10⁶ JIMT-1 or JIMT-1-BR cells in 50 μL of a 1:1 PBS/Matrigel mixture into the fourth mammary fat pad. Tumor growth was monitored twice weekly using digital calipers, and volume was calculated according to the modified ellipsoid formula, V = A × B^2^/2, where A and B represent tumor length and width, respectively.

For the experimental BrM model, mice received 1.75 × 10^5^ JIMT-1-BR cells suspended in 100 μL PBS by intracardiac injection into the left ventricle, as previously described [31]. Metastatic progression was monitored twice weekly by MRI using a BioSpec 9.4 T scanner (Bruker BioSpin) under 2% isoflurane anesthesia. Respiratory rate and body temperature were continuously monitored using a physiological monitoring system (SA Instruments). High-resolution coronal T2-weighted images were acquired using a TurboRARE sequence (TE = 33.01 ms, TR = 2500 ms, FOV = 20.0 × 20.0 mm, matrix = 256 × 256, five averages, 18 slices, 0.4 mm thickness; acquisition time, 6 min 40 s).

### Positron emission tomography imaging with [^89^Zr]Zr-DFO-trastuzumab

Mice bearing orthotopic breast tumors (*n* = 3 per cell line) or experimental BrM (*n* = 5) received approximately 7 MBq of [^89^Zr]Zr-DFO-trastuzumab intravenously in 100 μL PBS. PET imaging was performed 5 days after tracer administration under 2% isoflurane anesthesia using a prototype high-sensitivity small-animal PET scanner based on resistive plate chamber technology, providing an isotropic spatial resolution of 0.5 mm [37]. Throughout the imaging procedure, respiratory rate and body temperature were continuously monitored using a physiological monitoring system (SA Instruments).

MRI was subsequently acquired in the same position for anatomical co-registration. T2-weighted MRI was performed using a 2D TurboRARE sequence (TE/TR = 33/6164 ms, FOV = 80.0 × 45.0 mm, matrix = 320 × 320, three averages, RARE factor = 8, echo spacing = 11 ms, 60 sagittal slices, 0.5-mm thickness; acquisition time, 12 min 19 s).

PET data were analyzed using PMOD software v3.6 (PMOD Technologies) with the FUSION Tool. PET and MRI datasets were co-registered, and volumes of interest (VOIs) were delineated on PET images using the corresponding anatomical MRI images as a reference. Regions of interest (ROIs) were manually delineated around primary tumors and BrM lesions. Radiotracer uptake was quantified as the mean standardized uptake value (SUV_mean_), calculated by normalizing tissue radioactivity concentration (Bq/mL) to the decay-corrected injected activity (Bq) and each animal’s body weight. Following imaging, animals were euthanized and perfused with PBS. Primary tumors and selected organs were excised, weighed, and their radioactivity was measured using a CRC®−55tW automated well counter (Capintec). Biodistribution data were expressed as the percentage of injected dose per gram of tissue (%ID/g).

### Dynamic contrast-enhanced magnetic resonance imaging (DCE-MRI)

BBB permeability was assessed by DCE-MRI in mice bearing BrM (*n* = 5), as previously described [38]. Dynamic images were acquired using a DCE-FLASH sequence with the following parameters: TE = 2.5 ms, TR = 100 ms, FA = 70°, FOV = 20 × 20 mm, acquisition matrix = 156 × 156, 18 contiguous coronal slices (0.4 mm thickness), 60 dynamic acquisitions, seven signal averages, an acquisition time of 49.7 s per dynamic scan, and a total imaging time of 59 min 42 s. Five baseline dynamic acquisitions were obtained before intraperitoneal administration of the gadolinium-based contrast agent gadobutrol (Gadovist®; LUSAL). Image acquisition then continued for an additional 60 post-contrast dynamic scans to monitor the temporal distribution of the contrast agent. Image analysis was performed by semi-automatically delineating ROIs around each metastatic lesion, with corresponding ROIs defined in the contralateral non-tumor-bearing brain parenchyma. Changes in signal intensity were quantified over time for each ROI, allowing the evaluation of tissue perfusion and BBB permeability.

### Therapeutic evaluation of trastuzumab and [^177^Lu]Lu-DOTA-trastuzumab

Therapeutic efficacy of trastuzumab (Evidentic GmbH) and [^177^Lu]Lu-DOTA-trastuzumab was evaluated in mouse models of primary BC and BrM. In the orthotopic BC model, treatment began when tumors reached a mean volume of 35–60 mm^3^. Animals were randomly assigned to one of three treatment groups (*n* = 3 per group): (i) trastuzumab administered intravenously at a loading dose of 7.5 mg/kg on day 0, followed by maintenance doses of 3.5 mg/kg twice weekly (days 3, 7, 10, and 14); (ii) a single intravenous administration of [^177^Lu]Lu-DOTA-trastuzumab (8.0 ± 1.20 MBq, containing 60 μg trastuzumab); or (iii) vehicle control (0.9% NaCl). All injections were delivered in a final volume of 100 μL. Tumor progression was monitored twice weekly by MRI for up to 56 days. Animals were euthanized when tumor volume reached or exceeded 250 mm^3^ or upon meeting predefined humane endpoint criteria.

In the experimental BrM model, therapy was initiated following MRI confirmation of metastatic lesions with an average volume of approximately 0.33 mm^3^. Mice received intravenous saline (*n* = 3), trastuzumab (loading dose of 7.5 mg/kg on day 0 followed by 3.5 mg/kg on days 3, 7, 10, and 14; *n* = 3), or a single dose of [^177^Lu]Lu-DOTA-trastuzumab (8.0 ± 0.81 MBq containing 60 μg trastuzumab; *n* = 5). Body weight was recorded twice weekly, and animals were monitored daily for signs of discomfort or disease progression. Humane endpoints included body weight loss exceeding 20%, impaired mobility, or lack of responsiveness to external stimuli.

At the end of the study, or upon reaching humane endpoint criteria, mice were euthanized by cervical dislocation. Overall survival was evaluated using Kaplan–Meier method. Following euthanasia, primary tumors, metastatic lesions, and whole brains were collected, fixed in 4% paraformaldehyde, embedded in paraffin, and processed for subsequent histopathological evaluation.

### Statistical analysis

Data are presented as individual values or mean ± standard error of the mean (SEM), as specified in the corresponding figure legends. Graph generation and statistical analyses were performed using GraphPad Prism software. Comparisons between experimental groups were carried out using two-way analysis of variance (ANOVA) followed by Tukey's multiple comparisons post hoc test, as indicated in the respective figure legends. For two-way ANOVA, effect sizes were calculated as partial eta squared (ηp^2^) and interpreted according to Cohen's criteria. Survival distributions were analyzed using the Kaplan–Meier method and compared with the log-rank (Mantel–Cox) test. A *p* value < 0.05 was considered statistically significant. The value of n denotes the total number of independent biological replicates or animals, as indicated for each experiment.

## Supplementary Information

Supplementary Material 1.

## Data Availability

All data generated or analyzed in the study are available from the corresponding authors on reasonable request.
