# Supplementary material for: 177Lu-trastuzumab radionuclide therapy: a promising strategy for trastuzumab-resistant brain metastases in HER2+ breast cancer
Source: Mol Biomed. 2026 Sep 14;7:169. doi: 10.1186/s43556-026-00573-7 (PMC13575020; doi:10.1186/s43556-026-00573-7)
Supplement: Supplementary file 1 [file 43556_2026_573_MOESM1_ESM.docx]

**Supplementary materials for**

**^177^Lu-trastuzumab radionuclide therapy: a promising strategy for trastuzumab-resistant brain metastases in HER2+ breast cancer**

Liliana Santos^1,2,3,4^, Ivanna Hrynchak^3^, José Sereno^3^, Hugo R.S. Ferreira^1,3^, Magda Silva^3^, Alexandra Fonseca^3^, Rui Almeida^5^, Paulo Teixeira^5^, Antero J. Abrunhosa^3*^, Célia MF Gomes^1,2,4*^

^1^Faculty of Medicine, Institute of Pharmacology and Experimental Therapeutics, Coimbra Institute for Clinical and Biomedical Research (iCBR), University of Coimbra, Coimbra, Portugal;

^2^Center for Innovative Biomedicine and Biotechnology (CIBB), University of Coimbra, Coimbra, Portugal;

^3^Coimbra Institute for Biomedical Imaging and Translational Research, Institute for Nuclear Sciences Applied to Health (CIBIT/ICNAS), University of Coimbra, Coimbra, Portugal

^4^Clinical Academic Center of Coimbra (CACC), Coimbra, Portugal;

^5^Pathology Department, Centro Hospitalar e Universitário de Coimbra, Coimbra, Portugal.

*Corresponding authors:

Célia MF Gomes

Email: [cgomes@fmed.uc.pt](mailto:cgomes@fmed.uc.pt)

ORCID ID: 0000-0002-7497-4129

Antero Abrunhosa

Email: antero@pet.uc.pt

ORCID ID: 0000-0002-4145-854X

**Radiochemical stability tests**

For radiochemical stability analysis, the radiolabeled complexes [^89^Zr]Zr-DFO-trastuzumab and [^177^Lu]Lu-DOTA-trastuzumab were incubated in PBS and mouse serum at 37°C for 1, 24, 48, 72, 96, 120, 144, and 168 h. Following incubation, samples were spotted directly onto TLC paper strips, eluted with 0.1 M sodium citrate solution, and analyzed by TLC. This assay was performed in 3 independent experiments for each radiolabeled complex.

**Cell binding assays**

The HER2-binding affinity of [⁸⁹Zr]Zr-DFO-trastuzumab was evaluated using a radioligand cell-binding assay. The HER2-binding properties of [¹⁷⁷Lu]Lu-DOTA-trastuzumab were not directly assessed, as substitution of the radionuclide from ⁸⁹Zr to ¹⁷⁷Lu is not expected to alter the antibody’s binding characteristics. For the assay, HER2+ JIMT-1-BR and SUM190-BR cells were seeded in 6-well plates at densities of 3×10⁵ and 5×10⁵ cells per well, respectively, and allowed to adhere overnight. HER2-negative MDA-MB-231 cells were seeded at a density of 3×10⁵ cells per well and served as a negative control.

Subsequently, BC cells were incubated with 1 MBq/mL of [^89^Zr]Zr-DFO-trastuzumab in 2 mL of medium at 37°C for 0.5, 1, 2, and 3 h. After incubation, the medium was collected, and the cells were washed twice with ice-cold PBS. The cells were then lysed with 1 M sodium hydroxide for 5 min at RT. The radioactivity of all fractions (PBS, medium, and cell lysates) was measured using a γ-counter (Mirion Technologies, Capintec), and the percentage of [^89^Zr]Zr-DFO-trastuzumab bound to cells was calculated. This assay was performed in duplicate for each cell line in 3 independent experiments.

**Immunohistochemistry (IHC)**

Paraffin-embedded brain sections (10 μm) were deparaffinized and rehydrated through graded ethanol solutions. Antigen retrieval was performed in citrate buffer (10 mM, pH 6.0) using microwave heating (800 W) for 15 min. Tissue sections were then permeabilized with 0.5% Triton X-100, blocked with 5% BSA for 1 h at room temperature, and incubated overnight at 4°C with an anti-γH2AX primary antibody (1:100; Ser139; Abcam, ab81299). After washing, sections were incubated with Alexa Fluor 647-conjugated secondary antibodies (1:200; Invitrogen) for 1 h at room temperature. Nuclei were counterstained with Hoechst 33342 (5 μg/mL; Sigma-Aldrich) for 5 min in the dark. Finally, sections were mounted using Dako fluorescence mounting medium and imaged using an LSM 710 Meta confocal microscope (Carl Zeiss).

**Cresyl Violet Staining**

Paraffin-embedded brain sections (10 μm) were deparaffinized in xylene and rehydrated through a graded ethanol series to distilled water. Sections were stained with Cresyl Violet solution for 5–10 min, differentiated in 95% ethanol containing a small amount of acetic acid, dehydrated through graded ethanol solutions, cleared in xylene, and coverslipped using a permanent mounting medium. Stained sections were examined under bright-field microscopy to evaluate neuronal morphology, tissue architecture, and potential histopathological alterations, including neuronal loss and tissue necrosis.

**Histopathological analysis and HER2 immunostaining**

Primary tumor tissues and coronal brain sections containing metastatic lesions were sectioned at 4 µm and processed for hematoxylin and eosin (H&E; Sigma-Aldrich) staining or immunohistochemical detection of HER2. HER2 staining was performed using a rabbit monoclonal anti-HER2 antibody (Cell Signaling Technology, Cat. No. 2165). Antigen retrieval was carried out in EDTA–Tris buffer (pH 8.0) at 97 °C for 24 min, followed by incubation with a buffered protein-blocking solution to reduce nonspecific binding. Immunohistochemistry was performed on the Ventana Benchmark Ultra IHC/ISH automated staining platform using the OptiView DAB IHC Detection Kit (Ventana Medical Systems), a biotin-free multimer-based detection system, according to the manufacturer's instructions.

Stained sections were examined using a Nikon Eclipse 50i light microscope, and digital images were acquired with a Nikon Digital Sight DS-Fi1 camera. HER2 protein expression was evaluated by immunohistochemistry according to the College of American Pathologists (CAP) Template for Reporting Results of Biomarker Testing of Specimens from Patients with Carcinoma of the Breast (version 1.6.1.0, June 2025), which incorporates the interpretative criteria established in the 2018 American Society of Clinical Oncology/College of American Pathologists (ASCO/CAP) HER2 testing guidelines.**Fig. S1** Radiolabeling of trastuzumab with ^89^Zr and ^177^Lu results in stable complexes with high radiochemical purity. **a** Size exclusion high-performance liquid chromatography (SEC-HPLC) analysis of DFO-trastuzumab and DOTA-trastuzumab (left) and their corresponding ^89^Zr- and ^177^Lu-labeled constructs (right). **b** Radio-thin layer chromatography (radio-TLC) comparison of free ^89^Zr and ^177^Lu (left) with their respective trastuzumab-labeled constructs (right). (C) Radiochemical stability of [⁸⁹Zr]Zr-DFO-trastuzumab (left) and [¹⁷⁷Lu]Lu-DOTA-trastuzumab (right) assessed in PBS and mouse serum over 7 days.

**Fig. S2** Specific binding of [⁸⁹Zr]Zr-DFO-trastuzumab was assessed at different time points in HER2+ SUM190-BR and JIMT-1-BR brain metastatic breast cancer cells. HER2-negative MDA-MB-231 cells were used as a negative control. Data are presented as cell-associated radioactivity over time.

**Fig. S3** **a** Number of colonies formed by parental (JIMT-1 and SUM190) and brain-tropic (JIMT-1-BR and SUM190-BR) HER2+ breast cancer cells following treatment with increasing activities of [¹⁷⁷Lu]Lu-DOTA-trastuzumab. Colony formation was assessed 10–14 days after treatment, demonstrating a dose-dependent reduction in colony numbers in both parental and brain-tropic cells. Data are presented as individual values with mean ± SEM. **b** Quantification of comet tail length in parental and brain-tropic HER2+ breast cancer cells 5 days after treatment with increasing activities of [¹⁷⁷Lu]Lu-DOTA-trastuzumab. DNA damage is expressed relative to untreated controls (dashed line). Data are presented as mean ± SEM. Statistical significance was determined using two-way ANOVA followed by Tukey's multiple comparisons test. **P < 0.001, ***P < 0.0001 versus untreated controls.

**Fig. S4** **a.** Tumor uptake of [⁸⁹Zr]Zr-DFO-trastuzumab, expressed as mean standardized uptake value (SUV_mean_), in mice bearing JIMT-1 or JIMT-1-BR breast cancer xenografts 5 days after injection. **b.** *Ex vivo* biodistribution of [⁸⁹Zr]Zr-DFO-trastuzumab in major organs and tissues from the same mice 5 days post-injection. Tracer uptake was determined by gamma counting and expressed as the percentage of injected dose per gram of tissue (%ID/g). Data are presented as mean ± SEM (n = 3 mice per group).

**Fig. S5** Histopathological evaluation of brain tissue following [¹⁷⁷Lu]Lu-DOTA-trastuzumab treatment. **a.** Representative images of γH2AX immunofluorescence staining in brain metastatic lesions at low (20×) and high (63×) magnification. γH2AX-positive nuclei (red), indicative of radiation-induced DNA damage, were detected exclusively within metastatic tumor cells, whereas adjacent non-tumor brain parenchyma showed no detectable γH2AX signal. Nuclei were counterstained with Hoechst (blue). Scale bars: 100 μm (20×) and 20 μm (63×). **b.** Representative Cresyl Violet staining demonstrating preserved neuronal morphology and intact tissue architecture in the surrounding non-tumor brain parenchyma following treatment. The left panel shows a low-magnification view (40×), whereas the right panel shows a higher-magnification image (100×) of the boxed region.
